# Liver steatosis, selected organokines, and cardiovascular risk markers in rheumatoid arthritis

**DOI:** 10.3389/fendo.2026.1850882

**Published:** 2026-05-29

**Authors:** Mariusz Ciołkiewicz, Anna Kuryliszyn-Moskal, Ewa Jabłońska, Wioletta Ratajczak-Wrona, Jacek Janica, Włodzimierz Samborski, Piotr A. Klimiuk

**Affiliations:** 1 Department of Rehabilitation, Medical University of Bialystok, Bialystok, Poland; 2 Department of Immunology, Medical University of Bialystok, Bialystok, Poland; 3 Department of Radiology, Medical University of Bialystok, Bialystok, Poland; 4 Department of Rheumatology, Rehabilitation and Internal Diseases, Poznan University of Medical Sciences, Poznan, Poland; 5 Internal Medicine, Medical University of Bialystok, Bialystok, Poland

**Keywords:** BDNF, cardiovascular risk, FABP4, FGF21, left ventricular diastolic dysfunction, MASLD, NLR, rheumatoid arthritis

## Abstract

**Background:**

Metabolic dysfunction-associated steatotic liver disease (MASLD) is seen frequently in rheumatoid arthritis (RA); its often asymptomatic course delays diagnosis. Non-invasive scores and circulating biomarkers may enhance early detection. This study evaluated the prevalence of MASLD, the diagnostic performance of fatty liver index (FLI), hepatic steatosis index (HSI), and selected organokines (fatty acid binding protein 4, brain-derived neurotrophic factor, fetuin-A, and fibroblast growth factor 21) and explored associations of FLI and HSI with organokines, neutrophil-to-lymphocyte ratio, cardiovascular risk scores, and echocardiographic parameters of left ventricular diastolic dysfunction.

**Materials and methods:**

This cross-sectional, exploratory study included 51 patients with RA (46 women; mean age 48.8 ± 8.2 years; median disease duration 12 years). The diagnostic value of liver fat scores and organokines for MASLD was assessed by receiver operating characteristic curve analysis. Associations between liver fat scores and organokines, neutrophil-to-lymphocyte ratio, cardiovascular risk scores, and left ventricular diastolic dysfunction parameters were analyzed using univariable linear regression, while relationships with MASLD were evaluated by logistic regression.

**Results:**

MASLD was detected in approximately one-third of participants. FLI and fatty acid binding protein 4 demonstrated the best diagnostic discrimination for MASLD in patients with RA [area under the curve (AUC) values of 0.81 and 0.82; Youden index 0.52 and 0.56; cut-off 18.7 and 24.01 ng/mL; sensitivity 93% and 67%, specificity 58% and 89%, respectively]. FLI was positively associated with cardiovascular risk scores and left ventricular diastolic dysfunction parameters. Neutrophil-to-lymphocyte ratio was significantly associated with an increased MASLD risk (OR 2.53, p = 0.03).

**Conclusions:**

The FLI and HSI showed moderate-to-good discrimination for ultrasound-defined MASLD, with FLI yielding a numerically higher AUC but without statistically significant superiority over HSI. The FLI showed better performance than the HSI in reflecting cardiovascular risk and abnormalities in left ventricular diastolic function parameters. FABP4 and FGF21 were associated with MASLD-related measures and may warrant further investigation as adjunctive biomarkers. The low exploratory FLI threshold and the association between NLR and MASLD require confirmation in larger, externally validated cohorts using more accurate liver fat quantification and adequately adjusted models.

## 1 Introduction

Rheumatoid arthritis (RA) is a chronic autoimmune inflammatory disease characterized not only by cartilage destruction but also by frequent liver involvement, ranging from asymptomatic elevations in liver enzymes to steatosis, fibrosis, and even cirrhosis (1). Liver abnormalities occur in up to 44% of patients with RA, with the majority being asymptomatic, and may result from autoimmune processes, chronic systemic inflammation, primary biliary cholangitis, autoimmune hepatitis, or hepatotoxicity related to medications such as methotrexate and non-steroidal anti-inflammatory drugs (NSAIDs) (1–3). Immune-mediated mechanisms appear to influence liver function in RA independently of obesity or diabetes (4).

The prevalence of hepatic steatosis in patients with RA has been reported to range between 15% and 41.2% (2, 5–7). The risk of developing liver steatosis can be estimated using established indices based on clinical and biochemical parameters, such as the hepatic steatosis index (HSI) and the fatty liver index (FLI). According to the current guidelines of the European Association for the Study of the Liver (8), hepatic steatosis should primarily be diagnosed using non-invasive imaging methods, preferably ultrasonography. In 2023, non-alcoholic fatty liver disease (NAFLD) was redefined as metabolic dysfunction-associated steatotic liver disease (MASLD), introducing novel diagnostic criteria that emphasize the presence of cardiometabolic risk factors. This reclassification makes direct comparison with earlier studies using the NAFLD terminology challenging. When one or more cardiometabolic criteria [being overweight or obese or having dysglycemia or type 2 diabetes (T2D), elevated blood pressure, hypertriglyceridemia, and low high-density lipoprotein (HDL) cholesterol levels] are present, and daily ethanol intake does not exceed 20 g in women or 30 g in men, metabolic dysfunction-associated steatotic liver disease (MASLD) may be diagnosed.

Liver steatosis is associated with increased cardiovascular (CV) morbidity, mortality, and all-cause mortality (9–13). Hepatic dysfunction and CV pathology are mechanistically interconnected; pathological liver processes may contribute to systemic inflammation, cardiac remodeling, impaired myocardial energetics, and atherogenesis (14).

Hepatokines, liver-derived signaling molecules such as fetuin-A, coagulation factor XI, and selenoprotein P, play critical roles in thromboinflammation, endothelial dysfunction, microvascular injury, and cardiac remodeling. Elevated serum fetuin-A concentrations have been linked to an increased risk of CV disease independent of traditional cardiometabolic risk factors (15). Conversely, fibroblast growth factor 21 (FGF21) demonstrates cardioprotective effects in animal models and *in vitro* systems by improving myocardial energy efficiency and reducing cardiac hypertrophy and fibrosis. However, its markedly elevated circulating concentrations in patients living with obesity, MASLD, and heart failure likely reflect a compensatory stress response rather than an intrinsic cardioprotective effect (16). Data on the role of these markers in inflammatory arthritis remain emerging and require further investigation.

Several organokines are implicated in the pathogenesis of hepatic steatosis and T2D. Certain hepatokines, such as selenoprotein P and fetuin-A, promote hyperglycemia and insulin resistance; angiopoietin-like protein 3 inhibits lipoprotein lipase activity, while follistatin contributes to white adipose tissue insulin resistance, lipolysis, and excessive hepatic glucose production (15).

Accumulating evidence highlights complex interactions among hepatokines, adipokines, and myokines, modulated by nutritional status and physical activity, in the development of hepatic steatosis (17, 18). Adipokines derived from adipose tissue affect hepatic gluconeogenesis, lipid accumulation, and insulin sensitivity while promoting chronic low-grade inflammation and macrophage infiltration (19, 20). Fatty acid binding protein 4 (FABP4) was associated with liver steatosis in metabolic patients (21, 22). Myokines, such as brain-derived neurotrophic factor (BDNF), secreted by skeletal muscle during physical exercise, have demonstrated hepatoprotective effects through reduction of oxidative stress, inflammation, lipid peroxidation, and steatosis (23, 24). Population-based studies have shown an increased incidence of left ventricular diastolic dysfunction (LVDD) among patients with MASLD (25, 26); however, corresponding data in RA cohorts are lacking. In our previous work, we demonstrated associations between neutrophil-to-lymphocyte ratio (NLR), FGF21 levels, and echocardiographic markers of LVDD in patients with RA (27). In rheumatoid arthritis, MASLD has been linked to increased cardiovascular risk, but prior work has largely focused on the presence of MASLD as a binary diagnosis, hepatic indices or fibrosis scores as liver outcomes, or LV diastolic dysfunction as an isolated cardiac phenotype. However, the integrated relationship between MASLD diagnosed by simple hepatic indices, circulating organokines, and subclinical left ventricular diastolic dysfunction (LVDD) has not been systematically characterized in RA. Our study was specifically designed to address this gap. FABP4 and FGF21 were selected as key organokines tightly linked to hepatic steatosis and cardiovascular risk. Fetuin-A was included as a hepatokine connecting hepatic steatosis with insulin resistance and cardiometabolic risk, thus providing a complementary view of the liver–heart axis beyond FABP4 and FGF21. BDNF was chosen as a myokine with demonstrated hepatoprotective effects in experimental liver steatosis models.

The present study also aimed to determine whether FLI and HSI retain adequate ability to discriminate MASLD in RA and whether one of these indices better reflects RA-related drivers of steatosis (e.g., systemic inflammation, glucocorticoids, methotrexate use, and disease activity) beyond traditional metabolic factors.

## 2 Materials and methods

### 2.1 Study design

The present single-center, cross-sectional study aimed to assess the prevalence of MASLD, evaluate the diagnostic performance of liver fat scores (FLI and HSI) and selected organokines (FABP4, BDNF, fetuin-A, and FGF21), as well as analyze associations of the FLI and HSI with organokines, NLR, CV risk scores (Expanded Cardiovascular Risk Prediction Score for Rheumatoid Arthritis, ERS-RA; modified Systematic Coronary Risk Evaluation 2, mSCORE2), and echocardiographic parameters of LVDD in a cohort of patients with RA.

### 2.2 Population

The study included 51 patients with rheumatoid arthritis (RA) who fulfilled the 2010 ACR/EULAR classification criteria (28). The study protocol was approved by the Bioethics Committee of the Medical University of Bialystok, Poland (approval no. R-I-002/234/2016; 30 June 2016 and R-I-002/328/2017; 28 September 2017). A total of 55 participants were consecutively recruited between April 2017 and August 2019 at the Department of Rehabilitation, Medical University of Bialystok, Poland; of these, 2 declined to participate and 2 met exclusion criteria (1 overlap syndrome and 1 drug-induced liver disease); thus, 51 patients were included in the analysis. Available online calculators (ERS-RA, SCORE2, FLI, HSI, and FIB-4) were accessed in 2025, and the 2023/2024 MASLD criteria were retrospectively applied in the same year. All procedures were conducted in accordance with the Declaration of Helsinki, and written informed consent was obtained from all participants prior to enrolment. Apart from a confirmed diagnosis of RA, the inclusion criteria were being aged >18 years and having a stable treatment regimen for at least 3 months. Exclusion criteria were pregnancy, daily alcohol consumption >20 g in women or >30 g in men, overlap syndrome, viral hepatitis, autoimmune liver disease, drug-induced liver disease, active cancer, heart failure, advanced kidney disease, use of high-dose statins, and other medications with a significant impact on liver function. Participant flow is illustrated in **Supplementary Figure S1** in the **Supplementary Material**.

### 2.3 Clinical variables

Demographic and anthropometric data, including sex, age, height, and weight, were recorded. Body mass index (BMI) was calculated as follows = weight (kg)/height (m)^2^. Patients were classified according to WHO criteria as underweight (< 18.5 kg/m²), normal weight (18.5–24.9 kg/m²), overweight (25.0–29.9 kg/m²), or obese (grade I: 30.0–34.9 kg/m²; grade II: 35.0–39.9 kg/m²; grade III: ≥ 40.0 kg/m²).

Waist circumference (WC) was measured at the midpoint between the lower margin of the last palpable rib and the top of the iliac crest, at the end of a normal expiration with participants standing upright, feet together, arms at their sides, and abdomen relaxed. The average of two readings, differing by no more than 1 cm, was recorded. WC values ≥ 94 cm in men and ≥ 80 cm in women were classified as abnormal. Systolic and diastolic blood pressure measurements were obtained per current clinical recommendations,

Medical history encompassed RA duration, comorbid conditions, treatment regimen, daily alcohol intake, smoking status, and number of pregnancies. Hand and foot radiographs were evaluated, and disease activity was assessed using the Clinical Disease Activity Index (CDAI) (29). Participants’ functional disability level was measured using the modified Health Assessment Questionnaire (mHAQ) disability index. **Table 1** summarizes study participant characteristics.

**Table 1:** Study participant characteristics.

Variable	Mean ± SD/median (IQR)/n (%)
Age, years	48.80 ± 8.20
Sex, female	46 (90.2)
Daily alcohol intake, g	0.67 (0.00-1.33)
Smoking	25 (49)
Disease Duration, years	12 (4.95-20.25)
RF positivity	40 (78.4)
Erosive Disease	26 (50.9)
CDAI	25.00 ± 12.12
HAQ	0.60 (0.28-0.85)
mHAQ>0.5	20 (39.2)
Medication:	
Methotrexate	46 (90.2)
Leflunomide	1 (1.9)
Sulphasalazine	2 (3.9)
Cyclosporine	1 (1.9)
Arechine	1 (1.9)
Biologics	32 (62.7)
Corticosteroids	11 (21.6)
NSAIDS	28 (54.9)
Antihipertensive drugs	11 (21.6)
Statins	3 (5.7)
Body weight, kg	71.24 ± 15.66
Height, cm	164.00 (160.00-168.00)
BMI, kg/m^2^	26.30 ± 5.25
Waist circumference cm	89.67 ± 13.80
SBP mmHg	127.00 (121.00-146.00)
Comorbidites	
Hyperlipidemia	2 (3.9)
Hypertension	12 (23.5)
Type 2 diabetes	1 (1.9)
Gout	1 (1.9)
Asthma	2 (3.9)
Hashimoto disease	3 (5.9)
Chronic kidney disease	1 (1.9)
Antiphospholipid syndrome	1 (1,9)
Pregnancies	1.5 (0.00-2.00)

Data are presented as mean ± SD standard deviation or median and (IQR) interquartile range.

BMI, body mass index, CDAI, clinical disease activity index, HAQ, health assessment questionnaire, mHAQ, modified health assessment questionnaire, NSAIDS, non-steroidal anti-inflammatory drugs, RF, rheumatoid factor

### 2.4 Biochemical variables

Laboratory analyses included a complete blood count, AST (aspartate aminotransferase), ALT (alanine aminotransferase), GGT (gamma-glutamyl transferase), lipid profile parameters (total cholesterol), high-density lipoprotein cholesterol (HDL), low-density lipoprotein cholesterol (LDL), and triglycerides, estimated glomerular filtration rate (eGFR, calculated using the MDRD equation), rheumatoid factor, and high-sensitivity C-reactive protein (hsCRP) (Roche Diagnostics, Rotkreuz, Switzerland).

Serum concentrations of fatty acid binding protein 4 (FABP4) (Biovendor, Brno, Czech Republic), brain-derived neurotrophic factor (BDNF), fibroblast growth factor 21 (FGF21), and fetuin-A (R&D Systems, Minneapolis, USA) were measured strictly following the manufacturers’ instructions in samples stored at −80 °C using commercially available ELISA kits.

### 2.5 Liver fat scores

The hepatic steatosis index (HSI) was calculated using the formula: 8×(ALT/AST) + BMI + 2 (if type 2 diabetes) + 2 (if woman) (30). The online calculator (31) was accessed on 8 December 2025. HSI values below 30 indicated that MASLD can be ruled out, while values of 36 and above pointed to a positive diagnosis of MASLD.

BMI, WC, serum gamma-glutamyl transpeptidase, and triglyceride levels were used to calculate the fatty liver index (FLI) according to the following formula: FLI = [e ^0.953×loge (TG)+0.139×BMI+0.718×loge (GGT) +0.053×waist circumference−15.745^]/[1 + e ^0.953×loge (TG)+0.139×BMI+0.718×loge (GGT)+0.053×waist circumference−15.745^] × 100 (32) with the use of dedicated inline calculator (33) accessed on 8 December 2025. FLI values below 30 rule out MASLD; scores from 30 to below 60 are regarded as inconclusive, whereas scores of 60 and above indicate that MASLD is present.

### 2.6 MASLD diagnosis

Ultrasonographic diagnosis of liver steatosis was performed by a single experienced specialist with the use of Philips Epiq 7 (Bothell, Washington, United States). The sonographer was blinded to clinical data, laboratory results, and cardiovascular measurements at the time of image acquisition and interpretation. All examinations were conducted on the same ultrasound system, using a standardized protocol and predefined settings, in order to improve reproducibility. Hepatic steatosis was graded on B−mode ultrasound on a 4−point scale (0–3) based on liver echogenicity, hepatorenal contrast, and visualization of diaphragm and intrahepatic vessels, according to widely used criteria. Grade 0 indicated no hepatic steatosis, grade 1 mild, grade 2 moderate, and grade 3 severe steatotic changes.

According to available data, MASLD was diagnosed when steatosis was visualized on ultrasonographic imaging and one or more of cardiometabolic criteria was present: BMI ≥ 25 kg/m2, WC ≥ 94 cm in men and ≥ 80 cm in women, presence of T2D, blood pressure ≥ 130/85 mmHg or treatment for hypertension, plasma triglycerides levels ≥ 150 mg/dL, HDL-cholesterol ≤ 39 mg/dL in men and ≤ 50 mg/dl in women.

To rule out alternative causes of steatosis, patients with hepatitis B or C diagnosed by serologic testing (HBsAg and anti-HCV), known autoimmune liver disease or positive autoimmune serology compatible with autoimmune hepatitis, primary biliary cholangitis, or primary sclerosing cholangitis, drug-induced liver injury, and alcohol consumption >20 g/day (women) or >30 g/day (men) were not included in the study.

### 2.7 Liver fibrosis score

The FIB-4 (Fibrosis-4) index is a validated clinical score for estimating liver fibrosis, particularly in patients with HIV/HCV coinfection, and is calculated as follows: age×AST/(platelet count×ALT)age×AST/(platelet count×ALT​) (34). In our study, FIB-4 was computed using an online calculator (accessed 8 December 2025) (35). FIB-4 values <1.3 indicate a low risk of advanced liver fibrosis, values between 1.3 and 2.67 indicate an intermediate risk, and values >2.67 indicate a high risk of advanced fibrosis.

### 2.8 Cardiovascular risk scores

ERS-RA, dedicated to patients with RA (36), estimates 10-year CV morbidity incorporating conventional and RA-specific risk factors, which was calculated using a publicly available Excel macro (37). SCORE2 estimates 10-year fatal/non-fatal CV event risk per current guidelines of the European Society of Cardiology (38). For high-risk populations (Poland), a dedicated online calculator was used (39), with a 1.5 multiplier applied for RA (mSCORE2). SCORE2-DM was used for diabetic patients (40). All online calculators were accessed on 24 March 2025.

### 2.9 Echocardiographic assessment

Transthoracic echocardiography was performed by an experienced cardiologist using a Philips ClearVue 550 (Philips Healthcare, Best, Netherlands), following American Society of Echocardiography/European Association of Cardiovascular Imaging recommendations (41).

Left ventricular diastolic dysfunction parameters assessment included the following: left atrial diameter (LAD, parasternal long-axis), left atrial volume index (LAVI; apical 4-/2-chamber, modified Simpson’s method, indexed to body surface area), mitral inflow E/A ratio (pulsed-wave Doppler), septal/lateral e’ velocities (tissue Doppler), and E/e’ ratio (mean e’).

### 2.10 Statistical analysis

Analyses used R (version 4.4.2; the R Foundation for Statistical Computing, Vienna, Austria) and STATISTICA (version 13–3; StatSoft Polska, Kraków, Poland) with a significance level of α < 0.05. Continuous variables reported as mean ± SD or median(IQR) based on Shapiro-Wilk, skewness, kurtosis, and Levene’s tests. Two-step logistic regression analysis was employed to evaluate predictors of fatty liver (defined as MASLD = 1). Variable selection for the multivariable model was first based on univariable p-value < 0.25. In the second step, backward stepwise selection was performed to obtain the final model. Multicollinearity was evaluated using variance inflation factors. Model fit was assessed using Negelkerky R2.

To account for confounding while limiting model complexity and given the low number of outcome events, three pre-specified multivariable logistic regression models were fitted for each primary discriminative marker. The number of covariates included in each model was restricted to minimize the risk of overfitting. In Model 1, adjustment was performed for BMI only. In Model 2 adjustment included BMI, hypertension, and dyslipidemia, representing key metabolic risk factors. In Model 3 adjustment was performed using a composite confounder score, constructed by combining BMI, metabolic comorbidities (hypertension, dyslipidemia, and type 2 diabetes), disease activity (CDAI), and selected treatment exposures (glucocorticoids, DMARD therapy) in order to reduce dimensionality and avoid model overfitting.

To examine the impact of discriminative markers on liver fat scores, a linear regression approach was applied. Univariable linear regression with ordinary least square (OLS) estimation method was used to assess the association between FABP4 and FLI. Model assumptions were evaluated using graphical diagnostics and formal tests. Due to violations of normality of residuals, standard OLS inference was not considered fully reliable. In addition, non-parametric bootstrap resampling (5,000 iterations) was used to estimate standard errors, p-values, and 95% confidence intervals. Both percentile-based and bias-corrected and accelerated (BCa) bootstrap confidence intervals were calculated.

Diagnostic performance of markers for liver steatosis was evaluated using receiver operating characteristic (ROC) curves, determining the Youden index, cut-off values, sensitivity, specificity, and area under the curve (AUC). ROC curves were compared using DeLong’s test for correlated AUC values. Pairwise agreement between categorized CV risk scores was assessed using Cohen’s kappa statistic.

## 3 Results

### 3.1 Characteristics of the study population

The study enrolled 51 patients with rheumatoid arthritis (46 women, 5 men; mean age 48.80 ± 8.20 years; median disease duration 12 [IQR: 4.95–20.25] years). Mean CDAI was 25.00 ± 12.12, consistent with moderate disease activity, while median hsCRP was 2.88 (IQR: 1.06–6.19) mg/L, indicative of low inflammatory activity.

In our study, 98% of participants received conventional synthetic disease-modifying antirheumatic drugs (DMARDs), with 46 (90.2%) taking methotrexate at a mean weekly dose of 16.2 mg. Biological DMARDs were administered to 32 patients (62.7%), corticosteroids (mean prednisone equivalent: 6.2 mg/day) to 11 patients (21.6%), and NSAIDs to 28 patients (54.9%). Median daily alcohol intake was 0.67 g (IQR: 0.00–1.33); only one woman reached the maximum dose of 20 g/day.

### 3.2 Cardiometabolic risk factors

Of the participants, 3 (5.9%) were underweight, 20 (39.2%) were normal weight, 18 (35.3%) were overweight, and 10 (19.6%) were obese. Obesity was subclassified as follows: grade I in 6 patients (11.8%), grade II in 3 (5.9%), and grade III in 1 (1.9%). A total of 39 patients (76.5%; 35 women, 4 men) had abnormal WC.

Of the patients, 1 patient (1.9%) had T2D, and 12 (23.5%) had arterial hypertension. Elevated triglyceride levels were observed in 11 patients (21.6%), whereas reduced HDL cholesterol levels were found in 22 patients (43.1%; 19 women and 3 men).

### 3.3 Laboratory findings

Median AST, ALT, and GGT levels were 20 U/L (IQR: 15.00–25.00), 19.00 U/L (IQR: 15.00–32.00), and 10.90 U/L (IQR: 8.20–16.80), respectively, all within normal reference limits. Elevated liver enzymes were observed in 11 patients (21.6%). Specifically, one man (1.9%) had isolated GGT elevation, six women (11.8%) had elevated ALT, two women (3.9%) had elevated AST, and two women (3.9%) had elevations in both aminotransferases.

Median FABP4 and FGF-21 levels were 17.65 ng/mL (IQR: 13.24–24.18) and 17.65 pg/mL (IQR: 13.24–24.18), respectively. Mean BDNF and fetuin-A levels were 29.24 ± 13.29 ng/mL and 1371.14 ± 314.51 µg/mL, respectively.

### 3.4 Liver fat scores and ultrasonographic liver steatosis

An HSI value > 36 was observed in 28 patients (54.9%). An FLI cut-off ≥ 60 indicated hepatic steatosis in 10 patients (19.6%), whereas a cut-off ≥ 30 was indicative in 20 patients (39.2%).

Ultrasonography revealed liver steatosis in 15 patients (29.4%), comprising 10 with grade I and 5 with grade II. All 15 individuals with ultrasonographic steatosis fulfilled MASLD diagnostic criteria.

### 3.5 Echocardiographic findings

Mean peak E and A velocities, lateral and septal mitral annular velocities, E/A ratio, and median E/e’ ratio fell within normal reference ranges. Mean left atrial diameter (LAD), left atrial volume (LAV), and LAVI values indicated normal left atrial size. Laboratory findings, echocardiographic LVDD parameters, and CV risk scores are summarized in **Table 2**.

**Table 2:** Results.

Variable	Mean ± SD/median (IQR)
Total cholesterol, mg/dL	188.00 (159.50-206.50)
LDL, mg/dL,	93.41 ± 31.56
HDL, mg/dL	55.58 ± 16.32
TG, mg/dL	90.00 (73.00-139.50)
AST, U/L	20 (15.00-25.00)
ALT, U/L	19.00 (15.00-32.00)
GGT, U/L	10.90 (8.20-16.80)
FABP4, ng/mL	17.65 (13.24-24.18)
BDNF, ng/mL	29.24 ± 13.29
FGF21, pg/mL	17.65 (13.24-24.18)
Fetuin-A, µg/mL	1371.14 ± 314.51
hsCRP, mg/L	2.88 (1.06- 6.19)
Hemoglobin, g/dL	13.15 ± 1.25
eGFRml/min/1.73m^2^	92.08 ± 14.85
HSI	36.30 ± 6.82
FLI	23.80 (5.64-59.50)
FIB-4	0.87 (0.54-1.07)
E, cm/s	65.5 ± 13.2
A, cm/s	57.49 ± 13.87
E/A	1.20± 0.35
e’ lat, cm/s	13.25 ± 3.14
e’ med., cm/s	11.29 ± 2.45
E/e’	5.20 (4.60- 6.05)
LAD, mm	35.16 ± 4.77
LAV mL	43.1 ± 14.6
LAVI, mL/m^2^	23.82 ± 6.83
ERS-RA, %	4.10 (2.61-6.70)
mSCORE2, %	4.50 (2.85-8.25)

Data are presented as mean ± SD standard deviation or median and (IQR) interquartile range.

A, peak A-wave velocity, ALT, alanine aminotransferase, AST, aspartate aminotransferase, BDNF, brain-derived neurotrophic factor, E, peak E-wave velocity, e’ lat, lateral mitral annular velocity, e’med, septal mitral annular velocity, eGFR, estimated glomerular filtration rate, ERS-RA, expanded cardiovascular risk prediction score for rheumatoid arthritis, FABP4, fatty acid binding protein 4, FGF21, fibroblast growth factor 21, FLI fatty liver index, GGT, gamma-glutamyl transferase, HDL, high density cholesterol, hsCRP, high sensitivity C-reactive protein, HSI hepatic steatosis index LAD, left atrium long-axis diameter, LAV, left atrial volume, LAVI, left atrial volume index, LDL, low density cholesterol, mHAQ, modified health assessment questionnaire, mSCORE2, modified systematic coronary risk evaluation 2, TG, triglycerides.

### 3.6 Pairwise agreement between liver fat scores

**Figure 1:**
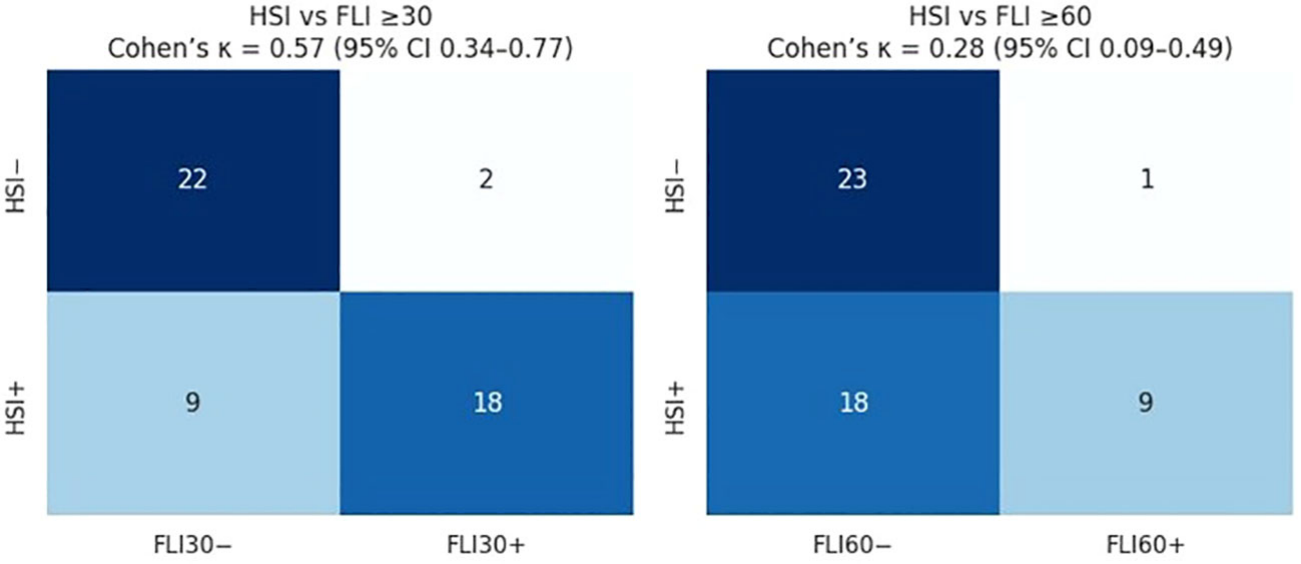
Pairwise agreement between liver fat scores.

Agreement between HSI and FLI was moderate at FLI ≥ 30 (κ = 0.57, 95% CI: 0.37-0.77) and fair at FLI ≥ 60 (κ = 0.28, 95% CI: 0.06-0.49), demonstrating threshold-dependent concordance in the RA cohort. **Figure 1** visualizes pairwise agreement between these liver fat scores.

### 3.7 Associations of liver scores with clinical parameters

#### 3.7.1 HSI discriminative markers

Each 1-year increase in age and each 1 mmHg increase in systolic blood pressure were associated with HSI increases of 0.35 (p = 0.002) and 0.16 (p < 0.001), respectively. These associations remained significant after adjustment for disease activity assessed by CDAI and DMARD exposure (age: β = 0.35, p = 0.004; SBP: β = 0.16, p < 0.001). No significant relationships were found between the HSI and RA treatments (MTX p = 0.79; biologics p = 0.56; steroids p = 0.16; NSAIDs p = 0.34). Among inflammatory markers, only NLR was positively associated (β = 2.27, p = 0.035); hsCRP was not significant (p = 0.14). There was no significant relationship between clinical disease activity as assessed by CDAI and HSI (p = 0.63).

#### 3.7.2 FLI discriminative markers

Female sex was a strong negative discriminative marker (β = -35.6, p = 0.009). Age (β = 1.51 per year, p = 0.002) and SBP (β = 0.59, p = 0.003) were positively associated. After adjustment for CDAI and DMARD exposure, these associations remained significant (age: β = 1.52, p = 0.004; SBP: β = 0.58, both p = 0.004). Among RA treatments, only steroid use was significantly negatively associated (β = -20.68, p = 0.04). NLR showed a strong positive association (β = 14.76, p = 0.001), while hsCRP showed no significant relationship (p = 0.63). No significant association was observed between CDAI and FLI (p = 0.85).

Both scores were positively associated with the number of pregnancies (HSI: β = 2.64, p < 0.001; FLI: β = 9.0, p = 0.004). Alcohol consumption showed no association (HSI p = 0.78; FLI p = 0.75).

### 3.8 Associations of liver steatosis indices with organokines

BDNF was positively associated with both scores (HSI: β = 0.22 per pg/mL, p = 0.002; FLI: β = 0.70, p = 0.03). No significant relationships were observed for fetuin-A (HSI p = 0.25, FLI p = 0.27). FGF21 was positively associated with the FLI (β = 0.03, p = 0.004) but not HSI (p = 0.24). FABP4 was positively associated with the HSI – every 1 ng/mL FABP4 increase raised the HSI by 0.11 (p = 0.009). In univariable analysis, higher FABP4 levels were associated with higher FLI values (β=0.41, standardized β=0.30). While the association reached statistical significance using classical OLS inference, violations of model assumptions prompted the use of bootstrap-based methods. Direction and magnitude of the association remain consistent using alternative approaches. Both percentile and BCa bootstrap methods supported a statistically significant association (p = 0.004 and p = 0.005, respectively). Sensitivity analysis is presented in **Supplementary Table 2** in the **Supplementary Material**.

### 3.9 Echocardiographic LVDD parameters and liver fat scores

In univariable analyses, the E/A ratio was negatively associated with both scores (HSI: β = -9.58; FLI: β = -52.58; both p < 0.001). Lateral e’ velocity showed negative associations (HSI: β = -0.69 per cm/s, p = 0.02; FLI: β = -4.31, p < 0.001), as did medial e’ (HSI: β = -1.25; FLI: β = -6.30; both p < 0.001). E/e’ showed only a trend toward positive association with FLI (β = 5.73, p = 0.08). Left atrial parameters were positively associated: left atrial diameter (HSI: β = 0.72 per mm, FLI: β = 3.06; both p < 0.001) and left atrial volume (HSI: β = 0.21 per mL, p = 0.001; FLI: β = 0.76, p = 0.007).

### 3.10 Associations with CV risk scores

FLI values were significantly positively associated with both ERS-RA (β = 1.59, p = 0.03) and mSCORE2 (β = 1.80, p = 0.005). No significant association was found between HSI and ERS-RA (p = 0.43), with only a trend toward a positive association with mSCORE2 (β = 0.26, p = 0.09).

**Figure 2:**
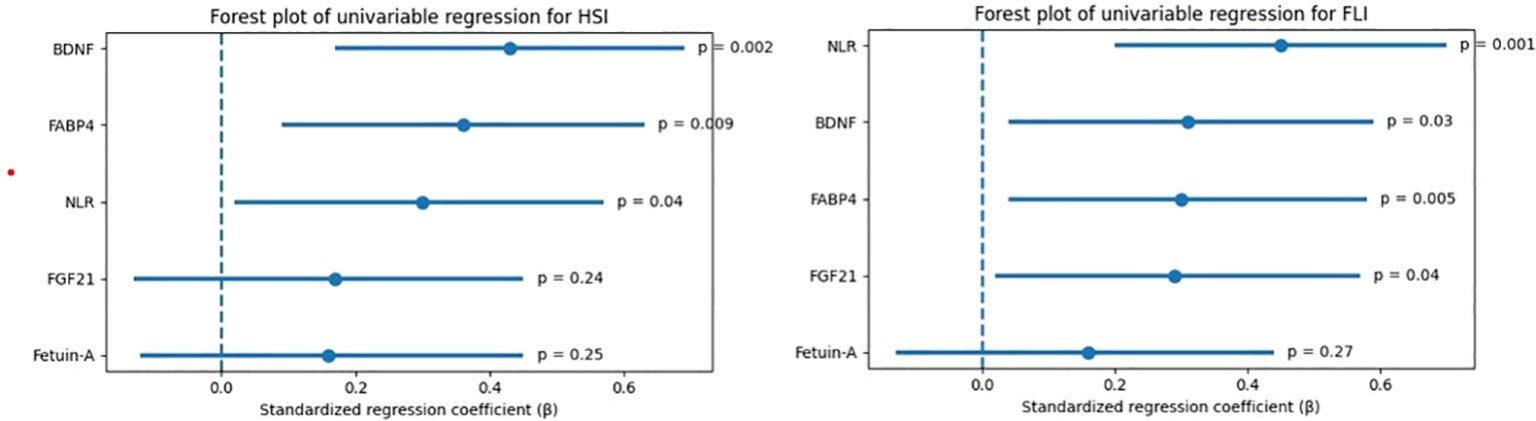
Forest plots of standardized β coefficients from univariable linear regression models for the hepatic steatosis index (HSI) and fatty liver index (FLI).

Results of univariable linear regression analyses for HSI and FLI are presented in **Supplementary Table 1** in the **Supplementary Material**. Forest plots of standardized β coefficients from univariable linear regression models for the HSI and FLI are presented in **Figure 2**.

### 3.11 Univariable logistic regression outcomes for MASLD

**Figure 3:**
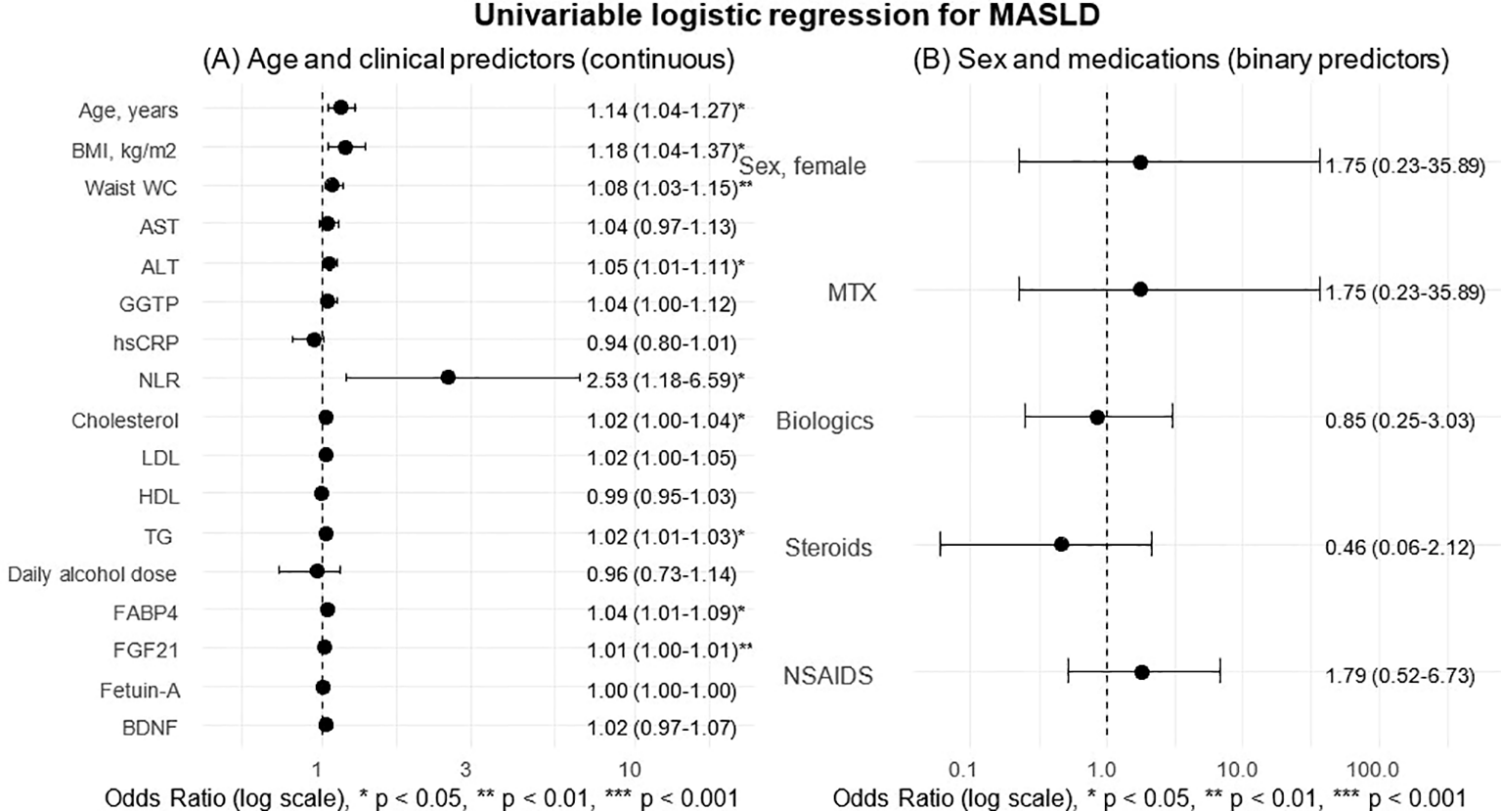
Forest plot presenting odds ratios for metabolic dysfunction-associated steatotic liver disease (MASLD) as outcomes of univariable logistic regression models **(A)** for continuous predictors and **(B)** for sex and medications (binary predictors).

In univariable logistic regression, higher ALT (OR 1.05 [95% CI: 1.01-1.11], p = 0.035), BMI (OR 1.18 per 1 kg/m² [95% CI: 1.04-1.37], p = 0.017), FABP4 (OR 1.04 [95% CI: 1.01-1.09], p = 0.03), FGF21 (OR 1.01 [95% CI: 1.00-1.01], p = 0.006), NLR (OR 2.53 [95% CI: 1.18-6.59], p=0.03), WC (OR 1.08 per 1 cm [95% CI: 1.03-1.15], p = 0.007), age (OR 1.14 per year [95% CI: 1.04-1.27], p = 0.01), total cholesterol (OR 1.02 [95% CI: 1.00-1.04], p = 0.034), and triglycerides (OR 1.02 [95% CI: 1.01-1.03, p = 0.013) were all significantly associated with higher odds of MASLD. Results of univariable logistic regression are summarized in **Table 3** and **Figure 3**.

**Table 3:** Descriptive statistics for analyzed parameters among patients with and without MASLD and verification of the univariable association between MASLD and analyzed parameters.

Variable	Patients with MASLD	Patients w/o MASLD	OR	95% CI	p
N	15	36	–	–	–
Age, years	53.67 ± 8.09	46.78 ± 7.45	1.14	1.04 to 1.27	0.011
Sex, female	14 (93.3)	32 (88.9)	1.75	0.23 to 35.89	0.630
RA duration	19.36 ± 10.59	9.5 (3.96-18.83)	1.07	1.01 to 1.13	0.033
BMI, kg/m^2^	29.80 (25.60-31.45)	24.10 (21.25-28.10)	1.18	1.04 to 1.37	0.017
WC	98.47 ± 10.58	86.00 ± 13.43	1.08	1.03 to 1.15	0.007
Methotrexate	14 (93.3)	32 (88.9)	1.75	0.23 to 35.89	0.630
Biologics	9 (60.0)	23 (63.9)	0.85	0.25 to 3.03	0.794
Steroids	2 (13.3)	9 (25.0)	0.46	0.06 to 2.12	0.364
NSAIDS	10 (66.7)	19 (52.8)	1.79	0.52 to 6.73	0.364
Daily alcohol dose	0.22 (0.00-1.33)	0.67 (0.00-1.33)	0.96	0.73 to 1.14	0.662
CDAI	26.37 ± 12.93	24.43 ± 11.91	1.01	0.96 to 1.07	0.602
AST	23.47 ± 6.20	20.92 ± 8.11	1.04	0.97 to 1.13	0.277
ALT	30.07 ± 15.07	21.08 ± 11.36	1.05	1.01 to 1.11	0.035
GGTP	15.50 (11.30-18.25)	10.10 (7.30-14.28)	1.04	1.00 to 1.12	0.163
Total cholesterol	196.00 (176.50-208.50)	176.00 (155.50-203.25)	1.02	1.00 to 1.04	0.034
LDL cholesterol	106.20 ± 33.64	88.09 ± 29.52	1.02	1.00 to 1.05	0.070
HDL cholesterol	53.42 ± 16.90	56.47 ± 16.24	0.99	0.95 to 1.03	0.540
TG	141.00 (87.50-198.50)	85.50 (70.00-108.00)	1.02	1.01 to 1.03	0.013
hsCRP	1.77 (1.17-5.54)	3.30 (0.99-7.46)	0.94	0.80 to 1.01	0.334
FABP4	24.89 (20.14-32.55)	14.33 (12.07-19.74)	1.04	1.01 to 1.09	0.030
FGF21	202.47 (116.20-680.84)	76.91 (40.75-131.00)	1.01	1.00 to 1.01	0.006
Fetuin-A	1500.41 ± 265.42	1317.28 ± 320.91	1.00	1.00 to1.00	0.065
NLR	2.29 ± 1.24	1.63 ± 0.62	2.53	1.18 to 6.59	0.033
BDNF	31.44 ± 9.37	28.32 ± 14.64	1.02	0.97 to 1.07	0.444
HSI	40.42 ± 6.57	34.59 ± 6.24	1.15	1.03 to 1.28	0.011
FLI	53.79 ± 28.57	9.15(4.54 36.45)	1.00	0.99 to 1.01	0.486
E/A	0.98 ± 0.28	1.29 ± 0.33	0.04	0.005 to.42	0.006
e’ lat, cm/s	12.30 (9.40-14.80)	13.51 ± 2.72	0.90	0.73 to 1.12	0.347
e’ med., cm/s	10.24 ± 2.17	11.72 ± 2.46	0.75	0.56 to 1.00	0.055
E/e’	5.30 (4.90-6.30)	5.19 ± 1.12	1.48	0.90 to 2.45	0.124
LAD, mm	36.67 ± 4.03	24.53 ± 4.96	1.11	0.97 to 1.27	0.148
LAV mL	45.05 ± 11.62	42.20 ± 15.73	1.01	0.97 to 1.06	0.537
LAVI, mL/m2	24.31 ± 4.93	22.46 (19.40-27.60)	1.02	0.93 to 1.11	0.734

### 3.12 Multivariable logistic regression model for MASLD

**Figure 4:**
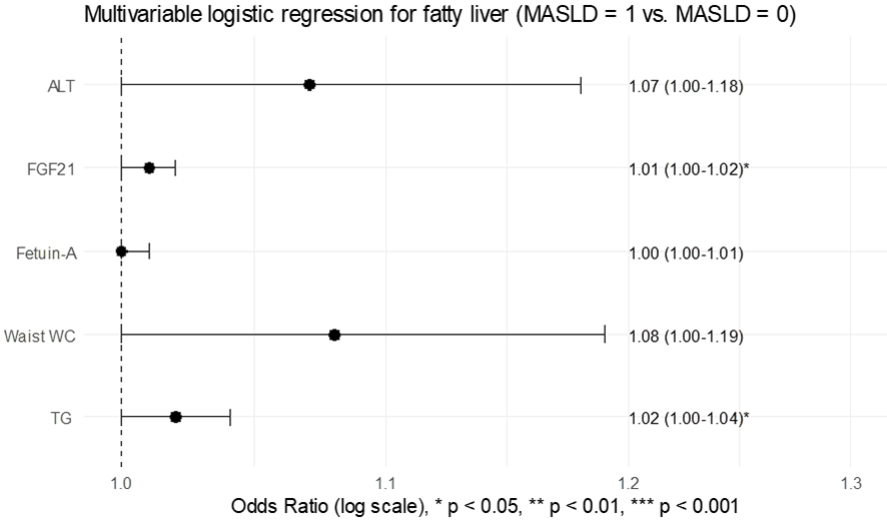
Forest plot presenting odds ratios for steatotic liver as outcomes of a multivariable logistic regression model.

A multivariable logistic regression model was fit to understand the optimal set of discriminative markers for fatty liver. It was confirmed that higher FGF21 was associated with increased odds of MASLD by 1% per one unit of FGF21 (OR = 1.01 [95% CI: 1.00-1.02], p = 0.02). Additionally, higher TG was associated with increased odds of MASLD (OR = 1.02 [95% CI: 1.00-1.04], p = 0.04). ALT and WC indicated positive association with higher odds of MASLD at the level of statistical trend (OR = 1.07 [95% CI: 1.00-1.18], p = 0.07 and OR = 1.08 [95% CI: 1.00-1.19], p = 0.09, respectively). The model also included Fetuin-A; however, a significant association was not confirmed (p = 0.18). Results of the multivariable logistic regression model for MASLD are summarized in **Figure 4** and in **Supplementary Table 3** of the **Supplementary Material**. Multivariable model fit measured with Negelkerky R2 was high and equal to 70%. Hosmer and Lemenshow GOF (Goodness of Fit) test resulted in p = 0.36, also indicating good model fit. Variance inflation factor indices were below 1.5 for all discriminative markers, confirming the lack of collinearity.

In multivariable analyses including confounders (**Supplementary Table 4**, **Supplementary Material**), FGF21 demonstrated a consistent and statistically significant association across all models. For FABP4, the association observed in univariable analysis was attenuated after adjustment; however, a trend toward significance was retained in models adjusting for BMI and the composite confounder score. In contrast, the association for NLR was not maintained after adjustment, while Fetuin-A and BDNF remained non-significant across all models.

### 3.13 ROC analysis for MASLD diagnosis

#### 3.13.1 Liver fat scores

The FLI showed very good discriminative value for MASLD (AUC = 0.81; Youden index = 0.52; cut-off = 18.7; sensitivity = 93%, specificity = 58%). The HSI performed slightly less well (AUC = 0.76; Youden index = 0.42; cut-off = 38.3; sensitivity = 67%, specificity = 75%).

#### 3.13.2 Organokines and NLR

Among organokines, FABP4 demonstrated the strongest diagnostic performance (AUC = 0.82, Youden index = 0.56) with a sensitivity of 67% and specificity of 89% at a cut-off of 24.01 ng/mL. FGF-21 showed good performance with AUC = 0.76 and Youden index = 0.47 (cut-off = 105.02 pg/mL; sensitivity = 80%, specificity = 67%). Fetuin-A performed less well (AUC = 0.70; Youden index = 0.45; cut-off = 1310 μg/mL; sensitivity = 87%, specificity = 58%). BDNF showed poor diagnostic value (AUC = 0.60). NLR had fair performance (AUC = 0.69; Youden index = 0.49; cut-off = 1.83; sensitivity = 80%, specificity = 69%).

**Figure 5:**
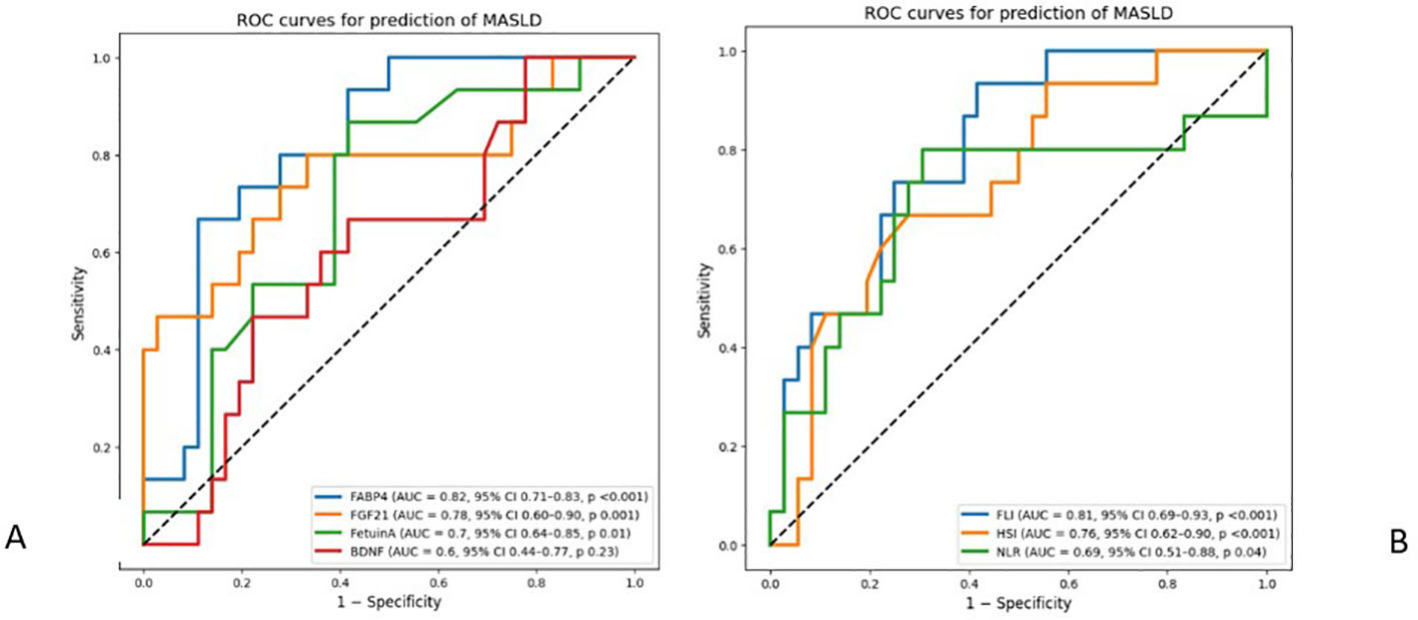
Receiver operating characteristic (ROC) curves for metabolic dysfunction-associated steatotic liver disease (MASLD) prediction **(A)** organokines, **(B)** NLR, and liver fat scores.

ROC curves for MASLD prediction showing AUC values, 95% CIs, and p-values for liver fat scores, organokines, and NLR are presented in **Figure 5**.

### 3.14 Comparison of ROC curves for fatty liver (MASLD = 1) for FLI and HSI

The discriminative performance of the FLI and HSI did not differ significantly. Although the FLI showed a higher AUC than the HSI (AUC 0.81 vs. 0.76), the difference was not statistically significant in DeLong’s test (Z = 1.14, p = 0.25). The 95% CI for the AUC difference ranged from -0.052 to 0.196, indicating no evidence of a meaningful advantage of either index in diagnosing fatty liver.

### 3.15 FGF21 and liver fibrosis score

Median FIB-4 in our cohort was low, at 0.87 (IQR 0.54–1.07), consistent with a low risk of advanced liver fibrosis in most patients. We did not observe a significant association between FGF21 and FIB-4 in the entire cohort (p = 0.14). In contrast, within the MASLD subgroup, we found a statistically significant positive association between FGF21 and FIB-4 (β = 0.0004, p = 0.044).

## 4 Discussion

The present study aimed to address the evidence gap regarding the utility of liver fat scores (HSI and FLI) and the role of organokines (FABP4, BDNF, FGF21, and fetuin-A) in hepatic steatosis among patients with rheumatoid arthritis.

### 4.1 Liver steatosis prevalence among patients with rheumatoid arthritis

In the present study, all patients with ultrasonographically confirmed liver steatosis met the MASLD diagnostic criteria, yielding a prevalence of 29.4%. This rate was nearly twice as high as that reported by Sellami et al. (2) in a cross-sectional study of 150 RA patients (mean age 57.1 years, mean RA duration 7.5 years) in which NAFLD prevalence was lower despite similar methotrexate (87%), higher NSAIDs (63%), and markedly higher oral corticosteroid exposure (88%). Our patients were younger, with longer disease duration. A meta-analysis by Mahapatro et al. (5) reported a pooled prevalence of NAFLD/nonalcoholic steatohepatitis (NASH) in patients with RA of 22.8%, varying by diagnostic method (FibroScan 33.3%, liver biopsy 43.9%, ultrasonography 30.5%), consistent with our observed MASLD prevalence. Erre et al. (6) reported an ultrasonographic steatosis prevalence of 38.7% among 223 RA patients with a higher mean age (61 years), shorter disease duration (7 years), similar BMI and WC, approximately 50% greater hypertension prevalence, reduced methotrexate use (81.1%), and higher corticosteroid exposure (36.3%). Similarly, Ausserwinkler et al. (7) reported a MASLD prevalence of 41.2% in 187 patients with RA, who were older, included more men (31%), and exhibited higher rates of T2D (7%), hypertension (35%), and obesity (33%). Notably, in that study, MASLD diagnosis was based on FLI values ≥ 60 plus at least one cardiometabolic risk factor rather than on ultrasonographic evidence.

### 4.2 Liver fat scores

In our patients with RA cohort, the FLI showed a numerically higher AUC than HSI, without statistical superiority for MASLD detection (DeLong’s test p = 0.25). This is in accordance with findings from the NHANES cohort (n = 5,524), where the FLI demonstrated superior, though not significantly different, performance for MASLD diagnosis (AUC 0.835) compared with the HSI (42). In that population, optimal cut-off values were 48.7 for the FLI and 38.3 for the HSI, while in our study, the corresponding thresholds were lower (18.7 and 38.3, respectively). Comparable findings were reported in a Chinese population cohort (n = 4,566), where the FLI and HSI achieved AUC values of 0.82 and 0.79, with optimal cut-offs of 22.4 and 32.9, respectively (43). Research among 1,300 adults with CT-confirmed MASLD also identified the FLI as the best discriminative marker (AUC 0.791, cut-off 29.9 (44).

In our RA population, the observed optimal FLI threshold for MASLD diagnosis was unexpectedly low (<30), a range typically considered to exclude hepatic steatosis. Given that the FLI formula incorporates BMI, WC, and triglyceride level, this finding suggests that not only metabolic but also inflammatory factors may contribute to hepatic steatosis in RA. This interpretation is supported by positive correlations between NLR and liver fat indices, as well as MASLD prevalence. Another potential explanation involves sarcopenia, a condition common among RA patients (24–30%) due to chronic inflammation, reduced physical activity, and corticosteroid exposure (45). Sarcopenic individuals may present with normal or slightly increased BMI values that mask low muscle mass and excess adiposity, both of which contribute to unfavorable metabolic effects. In our study, mean BMI was slightly elevated (26.3 kg/m^2^), but we lacked direct body composition data, including absolute/percentage body fat mass. Future research incorporating fat and lean mass assessment is warranted. Kim et al., in a prospective study of 10,030 South Korean adults followed for 16 years, demonstrated superior discriminative ability of the FLI versus HSI for metabolic syndrome development (AUC 0.85 vs 0.77) (46).

In our study, pairwise agreement between the HSI and FLI demonstrated threshold-dependent concordance and was moderate for FLI ≥ 30 and fair for FLI ≥ 60. Together with the mentioned FLI low cut-off value in ROC curve analysis, it may support the notion that lower FLI thresholds may improve MASLD detection in RA.

Furthermore, a retrospective nationwide Korean cohort study including 1,298,993 individuals aged 40 to 79 years, with a mean follow-up of 9 years, used FLI values ≥30 combined with at least one cardiometabolic risk factor to define MASLD, linking it to increased all-cause, cardiovascular, liver-related, cancer-related, and hepatocellular carcinoma-related mortality (11). Comparable associations were observed by Yan et al. in 6,074 prehypertensive/hypertensive patients, where MASLD, also defined as FLI values >30 and at least one cardiometabolic risk factor, predicted all-cause and CV mortality (10). Notably, in our cohort, only the FLI, but not the HSI, was significantly positively associated with CV risk scores (ERS-RA and mSCORE2). This likely reflects differences in score composition: the HSI incorporates ALT, AST, female sex, and T2D, whereas the FLI includes GGT, triglycerides, BMI, and WC—factors more directly linked to cardiometabolic risk. Accordingly, the FLI appears more informative than the HSI for assessing MASLD and CV risk in patients with RA.

### 4.3 FABP and liver steatosis

Evidence on the role of FABP4 in detecting liver steatosis among patients with RA is lacking. In a study on 389 high cardiometabolic risk individuals, in whom liver steatosis was diagnosed based on FLI values ≥ 60, FABP4 was associated with liver steatosis in metabolic patients (according to the novel definition - MASLD) (21). In our study, the FLI cut-off value for MASLD diagnosis was < 30, and FABP4 was associated with the FLI in the whole RA group.

Tanaka et al. also reported an independent association between FABP4 levels and MASLD in middle-aged and elderly Japanese individuals (defined as FLI ≥35 in men and ≥16 in women) (47). These findings, consistent with our results, suggest that FABP4 may contribute to the early identification of MASLD, even at subthreshold FLI ranges. Because FABP4 is involved in MASLD progression and may represent a potential therapeutic target (22), our findings regarding the role of FABP4 in RA could have important clinical implications.

### 4.4 FGF21 and liver fibrosis

In our study, we did not observe a relationship between FGF21 and liver fibrosis in the overall cohort. In contrast, within the MASLD subgroup, we found a statistically significant positive association between FGF21 and FIB-4, suggesting a potential contribution of FGF21 to liver fibrogenesis in this population. Future larger studies should validate the liver–heart axis in established fibrosis.

### 4.5 Limitations and strengths

This study has several limitations. First, the relatively small sample size (n = 51) and single-center design limit statistical power and generalizability. Second, the cross-sectional design precludes causal inference; prospective studies are needed to clarify temporal relationships. Third, liver steatosis assessment relied on conventional ultrasonography rather than advanced imaging modalities (magnetic resonance imaging, computed tomography, or transient elastography with controlled attenuation parameter). B-mode ultrasound has only moderate sensitivity for detecting mild steatosis, although specificity for more advanced steatosis is high, and performance is operator-dependent. This limitation may have led to under-detection of mild MASLD and misclassification in some participants, and we frame this as a potential source of bias that should be addressed in future studies with more advanced imaging confirmation. Fourth, obesity evaluation was based on BMI and WC without direct body composition analysis (bioimpedance or dual-energy X-ray absorptiometry). Fifth, according to the 2023 multi-society Delphi consensus, MASLD requires the presence of hepatic steatosis with cardiometabolic risk and the absence of other discernible causes of steatotic liver disease. In RA, long-term exposure to methotrexate, and other DMARDs, glucocorticoids, and NSAIDs may themselves promote hepatic steatosis and fibrosis, and we could not fully distinguish medication-induced or mixed-etiology steatosis from “pure” MASLD in individual patients. Our MASLD classification should therefore be interpreted as reflecting steatotic liver disease in patients with RA with metabolic dysfunction rather than definitive, etiologically pure MASLD, and residual misclassification bias cannot be excluded.

Given the lower optimal FLI cut-off values for MASLD diagnosis in patients with RA, this index may facilitate earlier detection and management of this common, often asymptomatic condition associated with increased mortality.

The diagnostic and prognostic value of FABP4, fetuin-A, FGF21, and BDNF for improving MASLD risk assessment, as well as their pathogenetic role and potential therapeutic utility in patients with rheumatoid arthritis and liver steatosis, warrants further investigation.

## 5 Conclusions

In this exploratory cross-sectional cohort of patients with rheumatoid arthritis, MASLD was detected in approximately one-third of participants.

The FLI and HSI showed moderate-to-good discrimination for ultrasound-defined MASLD, with the FLI yielding a numerically higher AUC but without statistically significant superiority over the HSI.

The FLI showed better performance than the HSI in reflecting cardiovascular risk and abnormalities in left ventricular diastolic function parameters.

FABP4 and FGF21 were associated with MASLD-related measures and may warrant further investigation as adjunctive biomarkers.

The low exploratory FLI threshold and the association between NLR and MASLD require confirmation in larger, externally validated cohorts using more accurate liver fat quantification and adequately adjusted models.

## Data Availability

The raw data supporting the conclusions of this article will be made available by the authors, without undue reservation.
