# Supplementary material for: Liver steatosis, selected organokines, and cardiovascular risk markers in rheumatoid arthritis
Source: Front Endocrinol (Lausanne). 2026 May 29;17:1850882. doi: 10.3389/fendo.2026.1850882 (PMC13259805; doi:10.3389/fendo.2026.1850882)
Supplement: Supplementary file 1 [file DataSheet1.docx]

Supplementary Material

# Supplementary Tables

**Table S1.** Results of univariable linear regression analyses for HSI and FLI

|  | **HSI** | | | **FLI** | | |
| --- | --- | --- | --- | --- | --- | --- |
|  | **β (95% CI)** | **Std β** | **p** | **β (95% CI)** | **Std β** | **p** |
| Sex, female | -3.63 (-10.1 to 2.8) | -0.16 | 0.26 | -35.55 (-61.86 to -9.24) | -0.36 | 0.009 |
| Age, years | 0.35 (0.13 to 0.57) | 0.42 | 0.002 | 1.51 (0.57 to 2.45) | 0 .42 | 0.002 |
| Pregnancy | 2.64 (1.23 to 4.05) | 0.51 | <0.001 | 9.00 (3.04 to 14.97) | 0 .43 | 0.004 |
| Methotrexate | 0.85 (-5.66 to 7.36) | 0.04 | 0.79 | 6.12 (-22.03 to 34.27) | 0.06 | 0.66 |
| Biologics | -0.44 (-1.92 to 1.05) | -0.09 | 0.56 | -2.49 (-11.51 to 5.10) | -0.11 | 0.44 |
| Steroids | -3.29 (-7.90 to 1.33) | -0.20 | 0.16 | -20.68 (-40.19 to -1.17) | -0.29 | 0.04 |
| NSAIDs | 1.93 (-1.01 to 2.87) | 0.14 | 0.34 | 8.70 (-4.02 to 12.73) | 0.15 | 0.30 |
| Alcohol g/24h | -0.08 (-0.63 to 0.48) | -0.4 | 0.78 | -0.38 (-2.78 to 2.02) | -0.05 | 0.75 |
| SBP, mmHg | 0.16 (0.08 to 0.24) | 0.47 | <0.001 | 0.59 (0.21 to 0.98) | 0.41 | 0.003 |
| hsCRP mg/dL | -0.09 (-0.22 to 0.03) | -0.21 | 0.14 | -0.13 (-0.69 to 0.42) | -0.07 | 0.63 |
| NLR | 2.27 (0.18 to 4.36) | 0.30 | 0.035 | 14 .76 (6.27 to 23.25) | 0 .45 | 0.001 |
| BDNF, pg/dL | 0.22 (0.09 to 0.35) | 0.43 | 0.002 | 0.70 (0.09 to 1.30) | 0 .31 | 0.03 |
| FABP4 | 0.11 (0.03 to 0.20) | 0.36 | 0.009 | 0.41 (0.04 to 0.78) | 0.30 | 0.03 |
| FGF21 pg/mL | 0.004 (-0.003 to 0.01) | 0.10 | 0.24 | 0.03 (0.002 to 0.06) | 0 .29 | 0.04 |
| Fetuin-A, µg/dL | 0.00 (-0.00 to 0.00001) | 0.16 | 0.25 | 0.00001(-0.00 to 0.00004) | 0.16 | 0.27 |
| E/A | -9.58 (-14.50 to -4.66) | -0.49 | <0.001 | -52.58 (-71.74, to -33.43) | -0.62 | <0.001 |
| e’ lat, cm/s | -0.69 (-1.27 to -0.11) | -0.32 | 0.02 | -4.31 (-6.71 to -1.92) | -0.46 | <0.001 |
| e' med., cm/s | -1.25 (-1.97 to -0.53) | -0.45 | <0.001 | -6.30 (-9.24 to -3.35) | -0.52 | <0.001 |
| E/e' | 1.61 (0.12 to 3.10) | 0.30 | 0.14 | 5.73 (-0.80 to 12.26) | 0.24 | 0.08 |
| LAD, mm | 0.72 (0.36 to 1.07) | 0.50 | <0.001 | 3.06 (1.51 to 4.60) | 0.49 | <0.001 |
| LAV, mL | 0.21 (0.09 to 0.33) | 0.45 | 0.001 | 0.76 (0.22 to 1.29) | 0.37 | 0.007 |
| ERS-RA | 0.14 (-0.20 to 0.48) | 0.11 | 0.43 | 1.59 (0.18 to 3.00) | 0.31 | 0.03 |
| mSCORE2 | 0.26 (-0.04 to 0.56) | 0.26 | 0.09 | 1.80 (0.57 to 3.03) | 0.42 | 0.005 |

Abbreviations: BDNF, brain-derived neurotrophic factor, e’ lat, lateral mitral annular velocity, e’med, septal mitral annular velocity, ERS-RA, expanded cardiovascular risk prediction score for rheumatoid arthritis, FABP4, fatty acid binding protein 4, FGF21, fibroblast growth factor 21, hsCRP, high sensitivity C-reactive protein, LAD, left atrium long-axis diameter, LAV, left atrial volume, mSCORE2, modified systematic coronary risk evaluation 2, SBP, systolic blood pressure

**Table S2**. Sensitivity analysis including outcome of univariable linear regression model for FLI as dependent variable and FABP4 as independent variable with alternative estimation methods

| **Estimation method** | **β** | **SE** | **95% CI** | **Std. β** | **p** |
| --- | --- | --- | --- | --- | --- |
| Classical OLS | 0.41 | 0.18 | 0.04 to 0.78 | 0.30 | 0.032 |
| Bootstrap (percentile CI) | 0.41 | 0.25 | 0.10 to 1.05 | 0.30 | 0.004 |
| Bootstrap (BCa CI) | 0.41 | 0.25 | 0.002 to 0.86 | 0.30 | 0.005 |

β – beta coefficient from regression model, SE – standard error, CI – confidence interval, std. β – standardized beta coefficient, OLS – ordinal least squares, BCa - bias‑corrected and accelerated

**Table S3.** Outcomes of multivariable logistic regression model for fatty liver

| Variable | OR | 95% CI | p |
| --- | --- | --- | --- |
| ALT | 1.07 | 1.00 to 1.18 | 0.073 |
| FGF21 | 1.01 | 1.00 to 1.02 | 0.022 |
| Fetuin-A | 1.00 | 1.00 to 1.01 | 0.176 |
| WC | 1.08 | 1.00 to 1.19 | 0.085 |
| TG | 1.02 | 1.00 to 1.04 | 0.044 |

OR – odds ratio as outcomes from multivariable logistic regression model for MASLD = 1 vs. MASLD = 0, CI – confidence interval.

Abbreviations: ALT, alanine aminotransferase, FGF21, fibroblast growth factor 21, TG, triglycerides, WC waist circumference.

**Table S4.** Outcomes of multivariable logistic regression for MASLD with adjustment to potential confounders, for primary focus parameters

| **Variable** | **Model 1: Logistic regression adjusted for BMI** | | | **Model 2: Logistic regression adjusted for BMI, hypertension and dyslipidemia** | | | **Model 3: Logistic regression adjusted for composite confounder score*** | | |
| --- | --- | --- | --- | --- | --- | --- | --- | --- | --- |
|  | **OR** | **95% CI** | **p** | **OR** | **95% CI** | **p** | **OR** | **95% CI** | **p** |
| FABP4 | 1.04 | 1.00-1.08 | 0.059 | 1.03 | 0.99-1.07 | 0.149 | 1.03 | 1.00-1.08 | 0.073 |
| FGF21 | 1.01 | 1.00-1.01 | 0.016 | 1.01 | 1.00-1.01 | 0.020 | 1.01 | 1.00-1.01 | 0.010 |
| Fetuin-A | 1.00 | 1.00-1.00 | 0.075 | 1.00 | 1.00-1.00 | 0.099 | 1.00 | 1.00-1.00 | 0.060 |
| NLR | 2.04 | 0.93-5.49 | 0.113 | 2.87 | 0.93-12.24 | 0.110 | 2.39 | 1.05-6.63 | 0.063 |
| BDNF | 0.99 | 0.94-1.05 | 0.841 | 0.97 | 0.91-1.03 | 0.380 | 1.00 | 0.95-1.05 | 0.978 |

OR – odds ratio as outcome from multivariable logistic regression model for MASLD = 1 vs. MASLD = 0, CI – confidence interval.

Abbreviations: BDNF, brain-derived neurotrophic factor, BMI, body mass index, FABP4, fatty acid binding protein 4, FGF21, fibroblast growth factor 21, NLR, neutrophil-to-lymphocyte ratio.

* Composite score was designed as sum of the following potential burdens: hypertension, dyslipidemia, diabetes type 2, use of DMARDS, use of glicosotocosteroids, CDAI>10, BMI≥25).

**Participant flow diagram**

RA patients assessed for eligibility (n = 55)

**Inclusion criteria** **Exclusion criteria**

age >18 years daily alcohol intake >20 g(W), >30 g(M),

stable treatment for 3 months overlap syndrome, viral hepatitis, autoimmune, drug-induced liver disease,

active cancer, heart failure,

advanced kidney disease,

high-dose statins/drugs impacting liver function

Excluded (n = 4)

2 declined to participate

2 met exclusion criteria

(1 overlap syndrome and 1 drug-induced liver disease)

Included in the study (n = 51)

Figure S1 Participant flow diagram
